# Supplementary material for: Anomaly Detection for Automated Data Quality Monitoring in the CMS Detector
Source: EPJ Res Infrastruct. 2026 Feb 9;10(1):4. doi: 10.1007/s41781-025-00147-2 (PMC12922165; doi:10.1007/s41781-025-00147-2)
Supplement: Supplementary file 1 [file 41781_2025_147_MOESM1_ESM.pdf]

Below are the members of the CMS Muon Detector Collaboration, organized by their institution.

RWTH Aachen University, III. Physikalisches Institut A, Aachen, Germany
Hebbeker Thomas
Reithler Hans
Schmidt Alexander
Merschmeyer Markus
Sharma Archana
Torres Da Silva Felipe
Hoepfner Kerstin
Eliseev Dmitry
Zaleski Shawn
Erdmann Johannes
Mausolf Florian
Spah Jan Lukas
Sarkisovi Valentina
Chinmay Seth
Daumann Caio
Ehlert Erik
Esper Nils

Academy of Scientific Research and Technology of the Arab Republic of Egypt, Egyptian Network of High Energy Physics, Cairo, Egypt
Ali Ellithi
Yasser Assran
Ahmed Abdelalim
H. Adballa
A. Radi

INFN Sezione di Bari, Universita di Bari, Politecnico di Bari, Bari, Italy
Gabriella Pugliese
Giuseppe Iaselli
Marcello Abbrescia
N. de Filippis
Colaleo Anna
Maggi Marcello
Nuzzo Salvatore
Ranieri Antonio
Simone Federica
Radogna Raffaella
Troiano Donato
Venditti Rosamaria
Verwilligen Piet
De Robertis Giuseppe
Licciulli Francesco
Loddo Flavio
Longo Luigi
Pellecchia Antonello
Ramos Lopez Dayron
M. Buonsante
W. Elmetenawee
N. Ferrara
A. Stamerra
A. Zaza
M. Ali
L. Generoso
F. Nenna

Benemerita Universidad Autonoma de Puebla, Puebla, Mexico
Isabel Pedraza
Cecilia Uribe Estrada
Felipe de Jesús Martínez Tapia
Epifanio PONCE
H.A.Salazar Ibarguen

INFN Sezione di Bologna, Universita di Bologna, Bologna, Italy
Navarria Francesco
Fabbri Fabrizio
Cavallo Francesca Romana
Dallavalle Gaetano-Marco
Marcellini Stefano
Rovelli Tiziano
Perrotta Andrea
Fanfani Alessandra
Masetti Gianni
Abbiendi Giovanni
Battilana Carlo
Primavera Federica
Lo Meo Sergio
Giacomelli Paolo
Guiducci Luigi
Fasanella Daniele
Lunerti Leonardo
Paggi Giulia
Farinelli Riccardo

CERN, European Organization for Nuclear Research, Geneva, Switzerland
Bianco Michele
Sharma Archana
Borgonovi Lisa

Panjab University, Chandigarh, India
Sushil Chauhan
Vipin Bhatnagar
Sunil Bansal
Bhawana Chauhan
Amarjeet Kumar RaM
Shiv Kumar
Kashish Verma
J. Babbar
A. Kaur
A. Kaur Sahota
H. Kaur
T. Sheokand
J. Singh

Centro de Investigacion y de Estudios Avanzados del IPN, Mexico City, Mexico
Heriberto Castilla Valdez
Ricardo Lopez Fernandez
Alberto Sanchez Hernandez
H. Crotte Ledesma

University of Colombo, Colombo, Sri Lanka
Upul Sonnadara
Kithsiri Jayananda Malagalage
Deshitha Wickramarathna
Kailasapathy, Balashangar

Institute of Experimental Physics, University of Debrecen
HUN-REN Institute for Nuclear Research
Szillasi Zoltan
Molnar Jozsef
Beni Noemi
Teyssier Daniel
Ujvari Balazs
Bencze Gyorgy
Zilizi Gyula

University of Delhi, Delhi, India
Kumar Ashok
Naimuddin M
B.C.Choudhary
M. Kumar Saini

Center for High Energy Physics (CHEP-FU), Fayoum University, El-Fayoum, Egypt
Mohammed Attia Mahmoud
I.Crotty

INFN Laboratori Nazionali di Frascati, Frascati, Italy
Luigi Benussi
Stefano Bianco
Davide Piccolo
Caponero Michele
Raffone Guido
Saviano Giovanna
Sabino Meola
Roberto Campagnola
Giulia Chiaraluce

Universiteit Gent, Gent, Belgium
Kirill Skovpen
Gül Gokbulut
Didar Dobur
Jules Vandenbroeck

Vrije Universiteit Brussel, Brussel, Belgium
Michael Tytgat
Yanwen Hong
Donya Ahmadi

Hanyang University, Seoul, Korea
Kim Tae Jeong
Asilar Ece
Ryou Yeonsu
J. Choi
J.A.Merlin

Institute for Research in Fundamental Sciences, Teheran, Iran
Mojtaba Mohammadi Najafabadi
Behzad Boghrati
Fateme Esfandi
Mohammad Ebrahimi
Elham Zareian
Mohammad Nourbakhsh

Saha Institute of Nuclear Physics, Kolkata, India
Majumdar Nayana
Mukhopadhyay Supratik
P. Rout

Korea University, Seoul, Korea
Hong ByungSik

Kyung Hee University, Department of Physics, Seoul, Korea
Goh Junghwan
Seungjin Yang
Jongwon Shin

Institut de Physique des 2 Infinis de Lyon, Villeurbanne, France
Gerald Grenier
Maxime Gouzevitch
Laurent Mirabito
Christophe Combaret
Galbit Geoffrey
Elise Jourd'hui
Jie Xiao
L.Balleyguier
X.Chen
I.B.Laktineh
A. Luciol
W.Tromeur

Centro de Investigaciones Energeticas Medioambientales y Tecnologicas (CIEMAT), Madrid, Spain
Cerrada, Marcos
Fouz, Maria Cruz
Josa, Maria Isabel
Puerta Pelayo, Jesus
De La Cruz, Begona
Fernandez Bedoya, Cristina
Fernández Ramos, Juan Pablo
Redondo, Ignacio
Moran, Dermot
Goy Lopez, Silvia
Alcaraz Maestre, Juan
Cepeda, Maria
Colino, Nicanor
Gonzalez Lopez, Oscar
Fernández del Val, Diego
Alcalde Martínez, Martín
González Hernández, Manuel
Manzanilla Carretero, Oliver
Martín Viscasillas, Elvira
Calvo, Enrique
Blanco Ramos, Luis Carlos
Navarro Tobar, Álvaro
Redondo Ferrero, David Daniel
Cuchillo Ortega, Jesus
Francia Ferrero, David
García Romero, Julián
Martínez Morales, Juan José
Paz Herrera, Rolando
Puras Sánchez, Juan Carlos
Sastre, Javier
Cuadrado Calzada, Sergio

Universidad Autonoma de Madrid, Madrid, Spain
de Trocóniz Jorge F

National Centre for Particle Physics, Universiti Malaya, Kuala Lumpur Malaysia
Zolkapli Zukhaimira

INFN Sezione di Napoli, Universita di Napoli 'Federico II', Napoli, Italy; Universita della Basilicata, Potenza, Italy; Universita G. Marconi, Roma, Italy
Salvatore Buontempo
Luca Lista
Pierluigi Paolucci
Fabozzi Francesco
Iorio Alberto Orso Maria
Leonardo Favilla
Rossi Biagio
Carlo Di Fraia
Francesco Confortini
A.Cagnotta,
F.Carnevali,
A. De Iorio

National Centre for Physics, Quaid-I-Azam University, Islamabad, Pakistan
H. Hoorani
Saleh Muhammad
Ashfaq Ahmad
Irfan Asghar
Mehar Ali Shah
Muhammad Ali

Sultan Qaboos University, Muscat, Oman
Amr Radi

Universidad de Oviedo, Instituto Universitario de Ciencias y Tecnologias Espaciales de Asturias (ICTEA), Oviedo, Spain
Fernandez Menendez Javier
Folgueras Santiago
Alvarez Gonzalez Barbara
Gonzalez Caballero Isidro
Palencia Cortezón Enrique
Soto Rodríguez, Alejandro
Vico Villalba, Carlos
Javier del Riego Badás
Ayllón Torresano, Jorge
C. Ramon Alvarez
V. Rodriguez Bouza
A. Trapote
Daniel Estrada Acevedo
Miguel Obeso Menendez
Javier Cuevas
Pelayo Leguina
Javier Prado

{INFN Sezione di Padova, Universita di Padova, Padova, Italy; Universita di Trento, Trento, Italy
Gasparini Fabrizio
Checchia Paolo
Meneguzzo Anna Teresa
Zumerle Gianni
Gasparini Ugo
Zotto Pierluigi
Carlin Roberto
Ronchese Paolo
Simonetto Franco
Margoni Martino
Gozzelino Andrea
Lai Nicolò
Bellato Marco
Benettoni Massimo
Gonella Franco
Gulmini Michele
Montecassiano Fabio
Passaseo Marina
Ventura Sandro
A.Bergnoli
A.Bragagnolo
G.Grosso
E.Lusiani
G.Maron
M.Migliorini
J.Pazzini
R.Rossin
A.Triossi
M.Zanetti
A.Zucchetta

INFN Sezione di Pavia, Universita di Pavia, Pavia, Italy
Paola Salvini
Alessandro Braghieri
Paolo Montagna
Cristina Riccardi
Ilaria Vai
Paolo Vitulo
Calzaferri Simone
Mario Pelliccioni

State Key Laboratory of Nuclear Physics and Technology, Peking University, Beijing, China
Sijin Qian
Ban Yong
Chen Zhou
Levin Andrew Michael
Li Qiang
Wang Dayong
Zhe Li
A.Agapitos
K.Wang

Universidade do Estado do Rio de Janeiro, Rio de Janeiro, Brazil
Alberto Santoro
Sandro Fonseca De Souza
Mauricio Thiel
Dilson de Jesus Damião
Helio Nogina
Eliza Melo Da Costa
M.Barroso Ferreira Filho
B.C. Ferreira
K.Mota Amarilo

Centro Brasileiro de Pesquisas Fisicas, Rio de Janeiro, Brazil
Gilvan Augusto Alves
Fabio Marujo da Silva
Eduardo Alves Coelho

University of Ruhuna, Department of Physics, Matara, Sri Lanka
Welathantri Dharmaratna
Nadeesha Wickramage
Liyanage Kalpanie
Perera Nimantha

Instituto de Física de Cantabria (IFCA), CSIC-Universidad de Cantabria Facultad de Ciencias, Santander
Calderon Alicia
Piedra Gomez Jonatan
Matorras Pablo

Seoul National University, Seoul, Korea
Inseok Yoon
Yang Unki
J.Choi
J.Kim

University of Seoul, Seoul, Korea
Lee Jason Sang Hun
Park Inkyu
Jeremie Merlin
Donghyun Kim
Woojin Jang
Ian Watson
J.Heo
D.Kang
Y.Kang
Y.Yang
S.Kim
B.Ko
S.Yang

Sungkyunkwan University, Suwon, Korea
Younghoon Lee
Intae Yu
Y.Jeong
M.R.Kim

Bulgarian Academy of Sciences, Inst. for Nucl. Res. and Nucl. Energy, Sofia, Bulgaria
Georgi Sultanov
Plamen Iaydjiev
Roumyana Hadjiiska
Aleksandar Aleksandrov
Milena Misheva
Mariana Shopova
Petar Danev

University of Sofia, Sofia, Bulgaria
Leandar Litov
Borislav Pavlov
Peicho Petkov
Anton Dimitrov
Anton Petrov
Elton Shumka

Sun Yat-Sen University, Guangzhou, Chin
Zhengyun You

Georgian Technical University, Tbilisi, Georgia
Lomidze Irakli
Bagaturia Iuri
Zviadi Tsamalaidze
O. Kemularia

INFN Sezione di Torino, Universita di Torino, Torino, Italy; Universita del Piemonte Orientale, Novara, Italy
Maselli Silvia
Mariotti Chiara
Amapane Nicola
Kiani Bilal
Umoret Giulio
De Remigis Paolo

Universidad de Antioquia, Medellin, Colombia
Álvarez Ruiz Jose David
Vanegas Nelson
Manuel Alejandro Rodríguez
Alexis Ruales
César Rendón
F.Ramirez

Universidad de Los Andes, Bogota, Colombia
Carlos Avila Bernal
Jose Reyes
Andres Leonardo Cabrera
Andres Florez
Diego Barbosa
J.Fraga

Universidad Iberoamericana, Mexico City, Mexico
Mateo Ramirez Garcia
Elsa Fabiola Vazquez
Dalia Lucero Ramirez Guadarrama
M.A.Shah
N.Zaganidis

Universite Libre de Bruxelles, Bruxelles, Belgium
De Lentdecker Gilles
De Bruyn Isabelle
Yang Yifan
Beshr Aya
Muhammad Ali
J.Jaramillo

Texas A&M University, College Station, Texas, USA
Safonov Alexei
Kamon Teruki
Patrick Flanagan
Gilmore Jason
Evaldas Juska
Hyunyong Kim
Muhammad Ahmad
Artem Bolshov
Yumeng Gao
Devin Aebi
Towsifa Akhter
O.Bouhali
R. Eusebi
T. Huang
M. Kizlov
S.Malhotra
R.Mueller
R.Rabadan
D.Rathjens

University of California, Los Angeles, California, USA
Hauser Jay
Saltzberg David
Abhisek Datta
Celia Lo
Robert Cousins
Vyacheslav Valuev
Mikhail Ignatenko
Ansar Iqbal
Nicholas Turner
Xiaofeng Yang
Samuel Crossley
J. Carlson
A. Dasgupta

University of California, Davis, Davis, California, USA
Erbacher Robin
Timothy Cox
Mani Tripathi
Jacob Steenis
Samantha Abbott
Sydney Ostrom
Richard Breedon
Tafoya, Juan
Hong Cai
Michael Mulhearn
Giovanni Mocellin
Ota Kukral
B. Regnery
Y.Yao

Wayne State University, Detroit, Michigan, USA
Karchin Paul

Florida Institute of Technology, Melbourne, Florida, USA
Hohlmann Marcus
Merrick Lavisnky
Butalla Stephen
Bandar Alsufyani
Erick Yanes

University of Wisconsin - Madison, Madison, Wisconsin, USA
Kevin Black
Camilla Galloni
Pieter Everaerts
Matthew Herndon
Susmita Mondal
He He
Abigail Warden
Anagha Aravind
Laurent Petre
A.Lanaro
R.Loveless
J.Madhusudanan Sreekala
D. Teague
W.Vetens
J. Marquez

University of California, Riverside, Riverside, California, USA
Robert Clare
M. Gordon
G. Hanson

University of California, Santa Barbara - Department of Physics, Santa Barbara, California, USA
Jeffrey Richman
Claudio Campagnari
Hualin Mei
Jaebak Kim
Prasanna Siddireddy
N. Amin
J.Bradmiller-Feld
T.Danielson
A.Dishaw
A.Dorsett
B.Marsh
M.Oshiro
F.Setti
M.F.Sevilla
S.Wang

Northeastern University, Boston, Massachusetts, USA
Emanuela Barberis
Darien Wood
David Morse
Yacine Haddad
Israr, Israr
Clark, Reily
Yixiao Han
Johan Bonilla Castro
Zheng, Junwen
G.Madigan
V.Nguyen

University of Florida, Gainesville, Florida, USA
Guenahk Mitselmakher
Andrey Korytov
Jian Wang
Vladimir Cherepanov
Neha Rawal
M. Dittrich
C. Aruta
Madorsky Alexander
Terentyev Nikolay
Barashko Victor
Ekaterina Kuznetsova
A.Muthirakalayil Madhu

Rice University, Houston, Texas, USA
Paul Padley
Karoly Banicz
Collin Arbour
J. Liu
M. Matveev

Institute of High Energy Physics of the Chinese Academy of Sciences, Beijing, China
Mingshui Chen
Dong, Xiaoli
Zebing Wang
W.Gong
Q.Hou
C.Jiang
H.Kou
Z.A.Liu
W.Luo
J.Song
L.Sun
N.Wang
Y.Wang
C.Zhang
Y.Zhang
H.Zhang
J.Zhao

College of Engineering and Technology, American University of the Middle East (AUM), Dasman, Kuwait
Yous Gharbia
Beyrouthy Taha
G.Alasfour
Y.Maghrbi
M.Otkur

Boston University, Boston, Massachusetts, USA
Indara Suarez
Frank Golf
Alexander Madorsky
Celia Fernández Madrazo
Javier Garcia de Castro
Samuel May
Kaitlin Salyer

The Ohio State University, Columbus, Ohio, USA
Stan Durkin
Christopher Hill
Darek Wenzl
Ryan De Los Santos
B.Bylsma
M. Carrigan

Instituto De Alta Investigacion, Universidad de Tarapaca, Arica, Chile
Shalini Thakur
Sumit Keshri

Authors affiliated with an international laboratory covered by a cooperation agreement with CERN

(Russia Dubna)
Vladimir Karjarvine
Victor Perelygin
Vladimir Palichik

Authors affiliated with an institute formerly covered by a cooperation agreement with CERN

(Russia St Petersburg)
Alexey Vorobyev
Victor Kim
Anatoli Egorov
Leonid Chtchipunov
Gennady Gavrilov
Victor Golovtcov
Mikhail Ivanov
Yury Ivanov
Petr Levchenko
Victor Murzin
Sergey Nasybulin
Vadim Oreshkin
Dmitry Sosnov
Igor Smirnov
Valentin Sulimov
Lev Uvarov
Sergey Vavilov
V.Ivantchenko

(Russia Tomsk)
A.Khodinov
V. Borshch
